# Experimental and Theoretical Analysis of Lead Pb^2+^ and Cd^2+^ Retention from a Single Salt Using a Hollow Fiber PES Membrane

**DOI:** 10.3390/membranes10070136

**Published:** 2020-06-30

**Authors:** Salwa Hadi, Ahmed A. Mohammed, Sama M. Al-Jubouri, Mahmood F. Abd, Hasan Shaker Majdi, Qusay F. Alsalhy, Khalid T. Rashid, Salah S. Ibrahim, Issam K. Salih, Alberto Figoli

**Affiliations:** 1 Environment Engineering Department, College of Engineering, University of Tikrit, Tikrit 34001, Iraq; env.salwa99@gmail.com; 2 Environment Engineering Department, College of Engineering, University of Baghdad, Baghdad 10071, Iraq; ahmed.abd@coeng.uobaghdad.edu.iq; 3 Department of Chemical Engineering, College of Engineering, University of Baghdad, Baghdad 10071, Iraq; sama.al-jubouri@coeng.uobaghdad.edu.iq; 4 Environment Geology Department, College of Science, University of Tikrit, Tikrit 34001, Iraq; dr.mahmood@tu.edu.iq; 5 Department of Chemical Engineering and Petroleum Industries, AlMustaqbal University College, Babylon 51001, Iraq; hasanshker1@gmail.com (H.S.M.); Dr_IssamKamil@mustaqbal-college.edu.iq (I.K.S.); 6 Membrane Technology Research Unit, Chemical Engineering Department, University of Technology, Baghdad 10066, Iraq; 80007@uotechnology.edu.iq (K.T.R.); salah.s.ibrahim@uotechnology.edu.iq (S.S.I.); 7 Institute on Membrane Technology, National Research Council (ITM-CNR), 87030 Rende (CS), Italy; a.figoli@itm.cnr.it

**Keywords:** hollow fiber membrane, CFSK model, CFSD model, CFFP model, wastewater treatment, heavy metals

## Abstract

The present work reports the performance of three types of polyethersulfone (PES) membrane in the removal of highly polluting and toxic lead Pb^2+^ and cadmium Cd^2+^ ions from a single salt. This study investigated the effect of operating variables, including pH, types of PES membrane, and feed concentration, on the separation process. The transport parameters and mass transfer coefficient (k) of the membranes were estimated using the combined film theory-solution-diffusion (CFSD), combined film theory-Spiegler-Kedem (CFSK), and combined film theory-finely-porous (CFFP) membrane transport models. Various parameters were used to estimate the enrichment factors, concentration polarization modulus, and Péclet number. The pH values significantly affected the permeation flux of the Pb^2+^ solution but only had a slight effect on the Cd^2+^ solution. However, Cd^2+^ rejection was highly improved by increasing the pH value. The rejection of the PES membranes increased greatly as the heavy metal concentration rose, while the heavy metal concentration moderately affected the permeation flux. The maximum rejection of Pb^2+^ in a single-salt solution was 99%, 97.5%, and 98% for a feed solution containing 10 mg Pb/L at pH 6, 6.2, and 5.7, for PES1, PES2, and PES3, respectively. The maximum rejection of Cd^2+^ in single-salt solutions was 78%, 50.2%, and 44% for a feed solution containing 10 mg Cd/L at pH 6.5, 6.2, and 6.5, for PES1, PES2, and PES3, respectively. The analysis of the experimental data using the CFSD, CFSK, and CFFP models showed a good agreement between the theoretical and experimental results. The effective membrane thickness and active skin layer thickness were evaluated using the CFFP model, indicating that the Péclet number is important for determining the mechanism of separation by diffusion.

## 1. Introduction

In the past few years, attention has been concentrated on the removal of heavy metal ions from wastewater due to their toxicity, and thus, their impact on human health. Therefore, according to environmental regulations, all of the heavy metals from wastewater of various industries must be removed, meaning that wastewater requires total control prior to its discharge into the environment [1]. Various traditional processes have been employed to remove heavy metals from effluents, such as chemical precipitation, electrocoagulation, solvent extraction, ion exchange, and adsorption on various sorbents, etc. Most of these above-mentioned processes suffer from economic limitations and other disadvantages. For example, both adsorption and ion exchange are inexpensive and highly effective for treating low concentrations of heavy metals; however, they generate hazardous sludge that requires regeneration or additional treatment, such as solidification. Furthermore, in the adsorption process, some adsorbents have limited capacities, low selectivity, require a long adsorption contact time, and show slow adsorption kinetics, even as most of the adsorbents, especially the synthetic nanoparticles, are expensive. Also, solvent extraction and chemical precipitation are considered to be polluting processes themselves [2,3,4,5,6,7,8,9,10,11,12].

Membrane separation processes have been found to be efficient, economic, and green (non-polluting) separation processes in comparison with the traditional and polluting methods mentioned above. Membrane separation processes have achieved wide use in treating various industrial wastewaters, with some used to remove heavy metals from wastewaters, such as reverse osmosis (RO), nanofiltration (NF), and ultrafiltration (UF) [13,14,15,16,17,18]. Nanofiltration membranes have mainly been used in various industries for the removal of heavy metals, compared with UF and RO processes, due to the high removal efficiency of NF and its ability to work at moderate pressure [18,19,20]. Separation by membranes generates concentrate/retentate that requires special attention before discharge into the environment. Also, the concentrate can accumulate at the membrane surface and reduce the water flow rate at a given transmembrane pressure. The literature shows that the method used to deal with concentrate mainly depends on its contents. The concentrate resultant from treating oily water can be reused for soap production. Also, the concentrate containing fructose, glucose, etc., can be used for the synthesis of beverages, while the concentrate resultant from treating wastewater containing heavy metals can be recycled for filling up the electroplating bath and rinsing purposes [21].

Much research has been conducted using the NF membrane process to remove heavy metal ions from wastewater, such as nickel, zinc, copper, arsenic, lead, cadmium, and chromium. Despite the efficient use of NF membranes in the removal of heavy metals, several factors have been found to determine the performance of NF membranes, including the membrane type, metal solution pH, metal ions, and metal concentration [19,20,21,22,23,24,25,26,27,28,29,30,31]. The literature rarely discusses the preparation of hollow fiber membranes with optimum specifications in the range located between NF and UF membranes that were applied for highly efficient heavy metal removal with high permeate flux. It is obvious that the main factors controlling the flow rate and the separation factor across the membrane are the characteristics of the membrane, such as the porosity, pore size, pore size distribution, and membrane thickness. Therefore, competition for the ability to manufacture hollow fibers has the best specifications, which makes the hollow fiber highly efficient for separation of solutes and mass production with approximately low costs. Because of the fact that the use of NF membrane in the process of removing heavy metals is common and the fact that NF membranes require high operating pressures compared to ultrafiltration membranes, three types of membranes whose specifications are between NF and UF have been prepared in this study. It is assumed that the efficiency of these hollow fibers is higher in terms of mass production and the removal rate of the heavy metals with those commercially manufactured membranes.

However, only a few studies have reported on highly concentrated solutions. Moreover, the investigation of various heavy metals using the same hollow fiber, at the similar operating conditions, provides important information about the appropriateness of the hollow fiber for a particular heavy metal under various conditions. Therefore, this work investigates the effective removal of two heavy metals (i.e., Cd^2+^ and Pb^2+^) from simulated aqueous solutions using three polyethersulfone (PES) membranes prepared for this purpose. These two heavy metals were selected due to their severe side effects on human health, whereas the removal efficiency of other heavy metals (e.g., Co^2+^) was studied in our previous work [32]. This research studies the effect of the operating conditions on the membrane separation performance, including pressure (1 bar), initial feed concentration (10–250) ppm, and pH solution (5.5–6.5). The literature dedicated to describing the concentration polarization and transport phenomena through hollow fiber membranes is not extensive. Therefore, this work aims to address this gap. So far, the membrane transport parameters and mass transfer coefficient have not been extensively studied using the following models: film theory, combined film theory-Spiegler-Kedem (CFSK), combined film theory-solution-diffusion (CFSD), combined film theory-finely-porous (CFFP) models, calculated concentration polarization model (CPM), enrichment factor (E_o_), and Péclet number (Pe). In previous literature, the membrane transport parameters and mass transfer coefficient were studied for only one membrane using a single selected model. But, this work will compare the membrane transport parameters, mass transfer coefficient, and the experimental results of the three different membranes using several selected models. 

## 2. Experimental Work

### 2.1. Materials and Methods 

Simulated wastewater was prepared by adding cadmium nitrate (Cd(NO_3_)_2_·4H_2_O) and lead nitrate (Pb(NO_3_)_2_) to distilled water. Several solutions were prepared with different concentrations of 10 to 250 ppm and pH values of 5.5 to 6.5. Three types of polyethersulfone (PES) membranes (PES type Radel) were provided by Solvay Advanced Polymers (Solvay, Brussels, Belgium), which were prepared using a dry/wet phase inversion method coded as PES1, PES2, and PES3 for heavy metal removal. The surface morphology and all the specifications of the PES membranes are summarized in Table 1, and details of the preparation method and measurement of the characteristics of the PES hollow fibers are presented elsewhere [33,34,35]. The reason behind selected different PES hollow fibers was to find the optimum specifications of the hollow fibers (e.g., pore size, porosity, and thickness) in the range located between NF and UF membranes that was applied for highly efficient heavy metal removal with high permeate flux. The pH values were measured using a calibrated pH meter (HQ411d, pH/mv, HACH Company, Loveland, CO, USA), whereas the concentrations of the metal ions in the simulated and treated solutions were measured using an AA-6200 atomic absorption flam emission spectrophotometer (Shimadzu Corporation, Kyoto, Japan) that was calibrated regularly, with the calibration curve verified before each sample set. The membrane surface charge depends on the pH value, with the membrane surface charge being negative for solution pH values higher than 5 and positive when pH values are less than 4 [30,31,32,33,34,35,36].

### 2.2. Membrane Filtration and Heavy Metal Rejection

**Figure 1:**
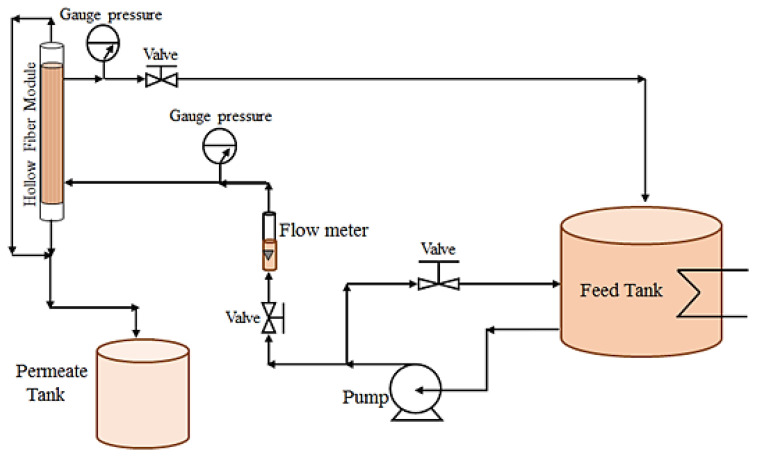
A schematic diagram of the membrane filtration test system.

The experiments testing the permeation flux of distilled water and heavy metal solutions as well as the rejection of heavy metals using PES1, PES2, and PES3 hollow fibers were achieved by module cross-flow pattern filtration. Two hollow fiber membrane modules composed of five fibers of the same membrane for each PES type were tested. Membrane experiments were conducted at a transmembrane pressure of 1 bar, heavy metal solution temperature of 25 °C, and metal solution concentration of 1000 ppm, using the PES hollow fiber membrane experimental setup shown in Figure 1. The effect of the pH and heavy metal concentration on the performance of three PES hollow fibers was studied extensively, and Table 2 shows the operating conditions of the membrane experiments. Permeation flux (J_V_) and heavy metal rejection (R%) were obtained from Equations (1) and (2), respectively:

J_v_ = V/t·A (1)

R (%) = [1 − C_p_/C_f_] × 100 (2)

where V is the permeate volume (l), *t* is the collected permeate time (h), *A* is the membrane surface area (m^2^), C_p_ is the concentration of the solute in the permeate, and C_b_ is an average bulk concentration of the solute in the feed (C_f_) plus what is in the concentrate/retentate (C_r_), estimated using Equation (3):

(3) $$C_{b}=\frac{C_{f}+{\;C}_{r}}{2}$$

After each set of experiments for a given feed concentration, the setup was rinsed with distilled water for 1 h at 4 bar pressure to clean the NF membrane experimental system. This was followed by measuring the pure water permeation flux with distilled water to ensure that the initial membrane flux was restored. Moreover, the pH value was adjusted using 1 M NaOH or 1 M HCl. By plotting the membrane flux (J_v_) versus the different applied pressures (ΔP), the membrane permeability (pure water permeability, L_p_) can be obtained from the slope of the line given by Equation (4):

(4) $$L_{p}=\frac{J_{v}}{\Delta P}$$

### 2.3. Models of Membrane Transport 

#### 2.3.1. Film Theory 

The concentration polarization (CP) phenomenon is known as the solute concentration accumulation at the surface of the membrane throughout the separation process. The solute is transferred by convection into the boundary layer and back by diffusion to the bulk solution [37,38].

**Figure 2:**
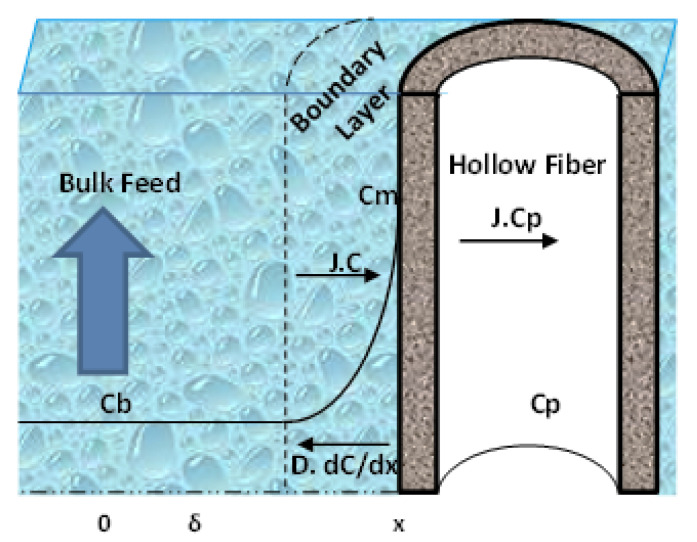
Schematic diagram of concentration polarization phenomenon in hollow fiber.

Figure 2 shows that the flux of the solute within the PES membranes decreased because of the CP phenomenon, where a gel layer formed on the membrane surface due to the retained solutes, resulting in an increase in osmotic pressure. From the basic principle of mass balance, the solute transfer at any point across the boundary layer can be described by Equation (5) [39]:

(5) $$\left(C-\mathrm{Cp}\right)J=D\;\frac{\mathrm{dc}}{\mathrm{dx}}$$

where D is the diffusivity of the solute, C is the concentration of the solute in the boundary layer, x is the distance from the membrane layer, and C_p_ is the concentration of the solute in the permeate solution. Equation (5) can be integrated with respect to the following boundary conditions to obtain Equation (6), based on the following:

$$C=C_{m}\;(\mathrm{at}\;x=0),\;C=C_{b}\;(\mathrm{at}\;x=\delta )$$

where C_m_ is the concentration of the solute at the surface of the membrane/water interface, C_b_ is the solute in the bulk solution, and δ is the edge of the mass transfer boundary layer,

(6) $$\frac{C_{m}-{\;C}_{p}}{C_{b}-{\;C}_{p}}=\exp(\frac{J}{k})$$

where k is the coefficient of mass transfer and is expressed as $k=\frac{D_{\mathrm{ab}}}{\delta }$, and D_ab_ is the diffusivity of solute a in water (solvent) b (cm^2^/s).

The typical expressions of the observed R_o_ and actual solute R are the rejections by a membrane given by Equations (7) and (8), respectively [40]:

(7) $$R_{o}=1-\frac{C_{p}}{C_{b}}$$

(8) $$R=1-\frac{C_{p}}{C_{m}}\;$$

Using Equations (7) and (8), Equation (6) can be rewritten in the following form [37]:

(9) $$\;\ln\left(\frac{1-R_{o}}{R_{o}}\right)=\frac{1}{k}\;J+\ln\left(P_{s}\right)$$

where $P_{s}=\frac{1-R}{R}$.

By plotting $\;\ln\left(\frac{1-\mathrm{Ro}}{\mathrm{Ro}}\right)\;$ versus J based on the experimental data, the overall permeability coefficient (P_s_) and the coefficient of mass transfer (k) can be calculated from the intercept of the line on the *y*-axis and the slope, respectively.

#### 2.3.2. Combined Film Theory/Solution-Diffusion Model (CFSD)

This model depicts the transfer mechanism, where the solute and solvent dissolve in the nonporous and homogeneous membrane surface, expressed by Equations (10) and (11) [41]:

(10) $$J=L_{p}\left(\Delta P-\Delta \pi \right)$$

(11) $$J_{s}=\left(\frac{D_{\mathrm{am}\;}K}{\delta }\right)\left(C_{m}-{\;C}_{p}\right)$$

where L_p_ is the water (solvent) permeability parameter that can be calculated from measurements of the Pure water permeation flux (PWP), and where $\frac{D_{\mathrm{am}}\;K}{\delta }$ is considered to be a single parameter, namely, the solute transfer variable or parameter.

(12) $$\frac{R_{o}}{1-R_{o}}=\left[\frac{J}{D_{\mathrm{am}}\;K/\delta }\right]\left[\exp\left(\frac{-J}{k}\right)\right]$$

Therefore, in the current study, the CFSD model will be described by Equation (12). The parameter $\frac{D_{\mathrm{am}\;}K}{\delta }$ and the coefficient of mass transfer (k) can be calculated numerically by inserting (R_o_) versus (J) data.

#### 2.3.3. Combined Film Theory-Spiegler-Kedem Model (CFSK)

An irreversible thermodynamics (IT) model can be applied in the absence of electrostatic interaction between the membrane and solute to explain the transfer of a single solute and solvent within a PES membrane, as reported by Kedem et al. [42]. The process is the sum of convective and diffusive fluxes, where these fluxes are due to the difference in pressure and to the concentration gradient at the membrane surface, respectively. In IT, the membrane is considered to be a black box. Therefore, the physicochemical properties of the membrane and solution system are considered as model parameters. The working equations of the nonlinear Spiegler-Kedem model [38,42] are as follows:

(13) $$J=-{\;L}_{p}\left(\Delta p-\sigma \Delta \pi \right)$$

(14) $$J_{s}=-\;P_{M}\Delta C_{s}+\left(1-\sigma \right)C_{s\;}J$$

Assuming the constant coefficients and constant fluxes P_M_ and σ, Equation (14) is integrated within the thickness of the membrane. The Spiegler-Kedem equation, which relates the retention of the solute (R, given by Equation (15)) with the volumetric flux of the water (solvent) and the permeability of the solute, results in the following:

(15) $$R=\frac{\sigma \left(1-F\right)}{1-\sigma F}$$

(16) where F=exp[−J a2]

(17) $$\mathrm{and}\;a2=\frac{1-\sigma }{P_{M}}$$

where σ, the reflection coefficient that assimilates the membrane rejection (e.g., σ=0), refers to a rejection with 0% and σ=1 denotes a solute rejection of 100%, $P_{M}$ is the permeability of the salt (L/m^2^·h), and  Lp is the membrane hydraulic permeability coefficient. F represents the flow parameter given by Equation (16). Additionally, Equation (15) can be rearranged to give Equation (18):

(18) $$\frac{R}{1-R}=a1\left(1-F\right)$$

(19) $$\mathrm{where}\;a1=\frac{\sigma }{1-\sigma }$$

Equation (20), shown below, is the result of substituting Equation (18) into Equation (9):

(20) $$\frac{\mathrm{Ro}}{1-\mathrm{Ro}}=a1\left[1-\exp\left(-\mathrm{Ja}2\right)\right]\left[\exp\left(\frac{-J}{k}\right)\right]$$

Equation (20) represents the CFSK model. The membrane σ, the $P_{M}$, and the coefficient of mass transfer (k) can be calculated by using a nonlinear parameter estimation method (SPSS version 22), where at different conditions, R_o_ versus J serve as inputs into the model [43].

#### 2.3.4. Combined Film Theory-Finely-Porous Model (CFFP)

The combined film theory-finely-porous model (CFFP) merges the effect of friction between the membrane pore wall and the solute molecules. The friction impact is taken into account as factor b. Equation (21) represents the working equation [44]:

(21) $$\frac{1}{1-R}=\left(\frac{b_{f}\varepsilon }{k}\right)+\left(\frac{k-b_{f}\varepsilon }{k}\right)\exp\left(-J\frac{\tau \varepsilon }{{\varepsilon b}_{f}D_{\mathrm{ab}}}\right)$$

where b_f_ is a factor measuring the friction between the membrane pore wall and the solute molecules, which is calculated from b_f_ = 1+ f_sm_/ f_sw_, where f_sm_ is the coefficient of friction between the membrane and the solute, whereas f_sw_ is the coefficient of friction between the solvent (water) and the solute.

The substitution of Equation (21) into Equation (9) results in Equation (22):

(22) $$\frac{R_{o}}{1-R_{o}}=\left(\frac{b_{f}\varepsilon }{k}-1\right)\left[1-\exp\left(-J\frac{\tau \delta }{{\varepsilon \;b}_{f}D_{\mathrm{ab}}}\right)\right]\exp\left(-\frac{J}{k}\right)$$

where

(23) $$b1=\left(\frac{b_{f}\varepsilon }{k}-1\right)$$

(24) $$b2=\frac{\tau \delta }{{\varepsilon \;b}_{f}D_{\mathrm{ab}}}$$

Equation (22) represents the CFFP model. A nonlinear parameter estimation method (SPSS version 22) can be used to calculate the membrane parameters and k by supplying, at various conditions, the data for R_o_ versus J for each set.

#### 2.3.5. Concentration Polarization Model (CPM) and the Enrichment Factor (E_o_) 

Concentration polarization (CP) is commonly characterized using the film theory model, where it is described by the boundary layer thickness across which the counter diffusion takes place. Here, the terms of the concentration in Equation (6) are substituted by the enrichment factors E (e.g., C_p_/C_b_) and E_o_ (e.g., C_p_/C_m_). Also, the CP in Equation (6) can be expressed by the Péclet number P_e_ (known as J/k), which produces Equation (24) [45]:

(25) $$\frac{1/E_{o}-1}{1/E-1}=\exp(P_{e})$$

Any increase or decrease in the concentration of the solute at the surface of the membrane compared to the bulk solution concentration determines the effect or range of the CP. The ratio C_m_/C_b_ represents the concentration polarization model (CPM) and is a perfect indication of the effect or range of the CP. No CP takes place when CPM ≤ 1. On the other hand, the model becomes increasingly neutralized when CPM > 1, at which point the CP’s impact on the selectivity and flux of the membrane becomes critical. Based on the definition of E_o_ and E, the CPM is equivalent to E_o_ and E, and using Equations (6) and (10), Equation (26) can be generated [43,45]:

(26) $$\frac{E}{E_{o}}=\frac{C_{m}}{C_{b}}=\frac{\exp\left(P_{e}\right)}{1+P_{e}\left[\exp\left(P_{e}\right)-1\right]}$$

Also, Equation (27) can be obtained by rearranging Equation (25) to calculate C_m_, as shown below [46]:

(27) $$\frac{C_{m}}{C_{b}}=\;(1-R_{o})+R_{o}\;\exp(J/k)$$

where, R_o_ = 1 − C_p_/C_m_.

The CP modulus can be lower or higher than 1, depending on the hollow fiber enrichment code E_o_. Equation (26) presents the parameters used to calculate the CP value: the boundary layer thickness *δ*, the hollow fiber enrichment E_o_*,* the volumetric flow rate through the hollow fiber J, and the coefficient of solute diffusion throughout the boundary layer fluid D. The boundary layer thickness *δ* is the most significant parameter that affects the CP. When *δ* decreases, Equation (26) indicates that the CP modulus is exponentially low. Thus, the optimal method for reducing the CP is to decrease *δ* by accelerating the turbulence around the surface of the hollow fiber [47]. Also, the CP is affected by the actual enrichment (E_o_) of the hollow fiber. For example, E_o_ equals 1 if the hollow fiber is completely unselective. The concentration gradient in the boundary layer does not take place unless there are changes in the species concentrations of the permeating solution across the hollow fiber. Moreover, when the difference in the permeability of the species increases, the actual enrichment E_o_ of the fiber is enhanced, and the concentration gradient formed at the boundary layer increases. Another significant characteristic of Equation (26) is that the E_o_ created by the fiber, not the actual selectivity α, determines the CP modulus and the fiber separation performance. Equation (26) demonstrates that increasing the total volumetric flowrate J within the hollow fiber increases the CP exponentially.

#### 2.3.6. Comparison of the Experimental Results (S^2^) and Model Predictions 

This study investigated the models’ validity and the type of fitting used by calculating the nonlinear parameters expressed by Equation (28) [46]:

S^2^ = ∑ (R_exp_ − R_th_)^2^/R_th_ (28)

where R_exp_ and R_th_ are the experimental and theoretical rejection of the solute respectively, as estimated by the models. If R_exp_ > R_th_, S^2^ will be large, while if R_exp_ ≤ R_th_, S^2^ will be small.

#### 2.3.7. Calculation of the Péclet Number (Pe)

In the study of transport phenomena in fluid flows, the Péclet number (P_e_) (defined as a dimensionless number) is considered to be a significant parameter. It corresponds to the ratio between the convective transfer J of a physical quantity and the flow and diffusive transfer k (D_ab_/δ) of the similar quantity driven by a proper difference. By Equation (29), the P_e_ is defined as follows:

(29) $$P_{e}=\frac{\mathrm{advective}\;\mathrm{transport}\;\mathrm{rate}}{\mathrm{diffusive}\;\mathrm{transport}\;\mathrm{rate}}\;i.e.,{\;P}_{e}=\frac{J}{k}$$

where k denotes the coefficient of mass transfer from the CFSK model [43].

## 3. Results and Discussion 

### 3.1. Effect of the Feed pH on the Membrane Performance

**Figure 3:**
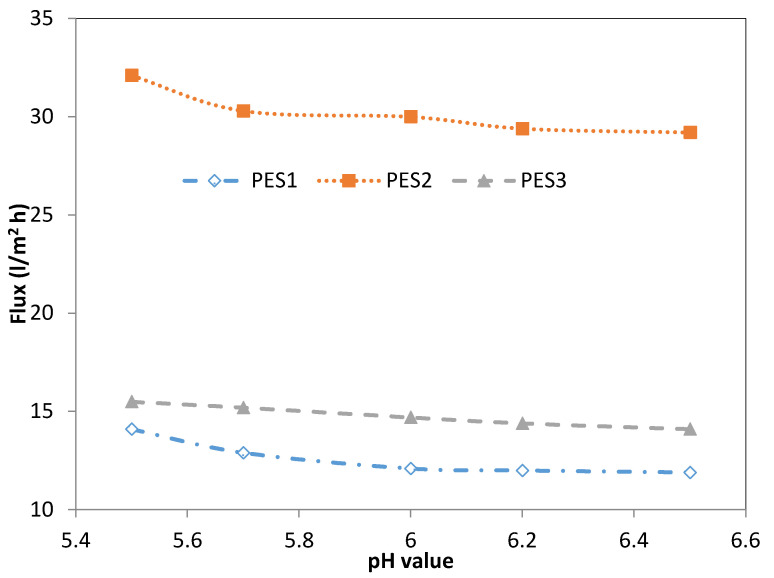
Effect of feed pH value on final permeate flux of PES1, PES2, and PES3 membranes for Pb^2+^ (experimental conditions: Pb^2+^ concentration of 100 ppm, transmembrane pressure of 1 bar).

**Figure 4:**
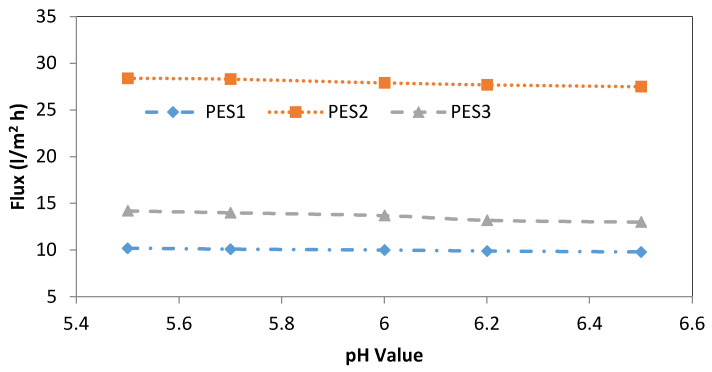
Effect of feed pH value on final permeate flux of PES1, PES2, and PES3 membranes, (experimental conditions: Cd^2+^ concentration of 100 ppm, transmembrane pressure of 1 bar).

Figure 3 and Figure 4 show the effect of the feed pH value on the permeation flux of the three types of PES membranes (i.e., PES1, PES2, and PES3) for 100 ppm lead and cadmium solutions at a transmembrane pressure of 1 bar and temperature of 25 °C. The permeation flux of the Pb^2+^ solution decreased from 14.1 to 12.1 (L/m^2^·h) when increasing the feed pH value from 5.5 to 6.0 using PES1, while no significant decrease in the permeation flux was observed when increasing the pH value to 6.5, as shown in Figure 2. Using PES2, the permeation flux for the Pb^2+^ solution decreased from 32.1 (L/m^2^·h) at a feed pH of 5.5, to 30.3 (L/m^2^·h) at a feed pH of 6.5, as shown in Figure 3. Moreover, a similar trend was obtained using PES3 to separate the Pb^2+^ solutions, as shown in Figure 3. The permeation flux slightly and gradually decreased from 15.5 to 14.1 (L/m^2^·h) with increases in the pH of the Pb^2+^ solution from 5.5 to 6.5. Regarding the Cd^2+^ solution, a trend similar to that of Pb^2+^ was observed for Cd^2+^, as shown in Figure 4, where the permeation flux slightly decreased from 10.2 to 9.8 (L/m^2^·h) as the pH value rose from 5.5 to 6.5 using PES1. In contrast, the permeation flux at pH 5.5 was 28.4 (L/m^2^·h), and it decreased slightly to 27.5 (L/m^2^·h) at a feed pH of 6.5 using the PES2 membrane, as shown in Figure 3. For PES3, the permeation flux decreased from 14.2 to 13 (L/m^2^·h) as the Cd^2+^ solution pH rose from 5.5 to 6.5, as shown in Figure 4. From the above results, it can be concluded that the pH value has a similar effect on the permeation flux of all membrane types at various pH values. This phenomenon is mainly attributed to the charge of the membrane surface. From a pH of 5.5 to 6.5, the charge of the membrane becomes more negative due to the increase in OH^-^; therefore, the adsorption of heavy metal ions occurs at the surface of the hollow fiber membrane because of the electrostatic attraction, which in turn led to a decrease in pore size of the membrane, thus decreasing the permeation flux and increasing the heavy metal rejection. The variations in the permeation flux as a result of changing the pore size of the hollow fiber with pH may be attributed to three reasons, first: expansion or contraction associated with a network of polymer membrane, second: electric-viscous effect, and third: net driving force higher than the osmotic pressure on the membrane surface [48]. Another explanation is due to shrinkage of the membrane layer as a result of differences in the hydration of membrane-ionized groups [26]. The same behavior was found by Tanninen et al. [30].

**Figure 5:**
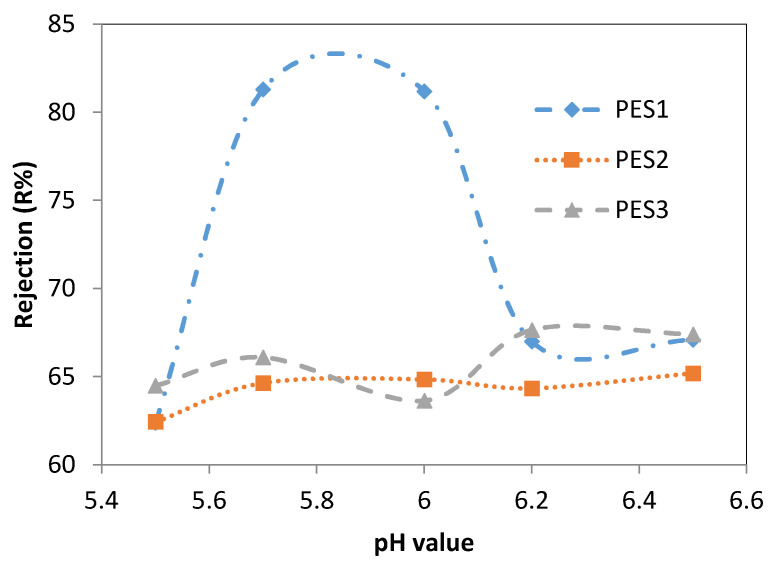
Effect of feed pH value on pb^2+^ rejection of PES1, PES2, and PES3 membranes (experimental conditions: pb^2+^concentration of 100 ppm, transmembrane pressure of 1 bar).

**Figure 6:**
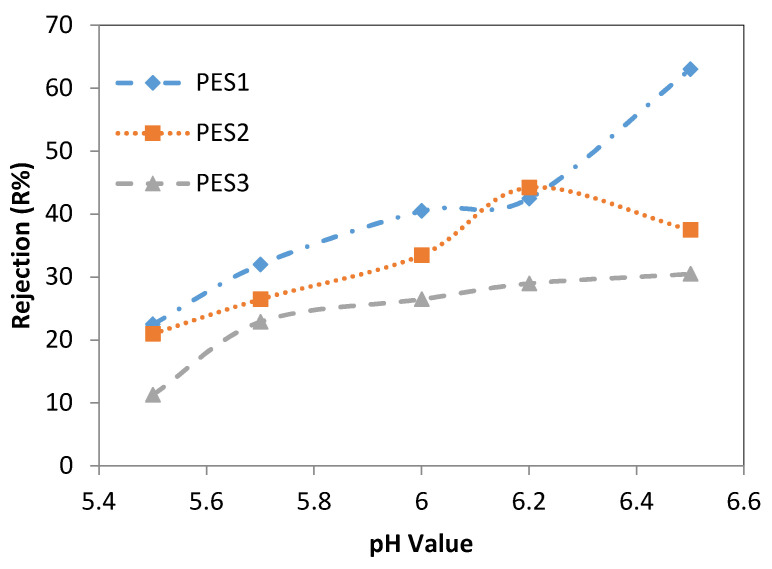
Effect of feed pH on Cd^2+^ rejection of PES1, PES2, and PES3 membranes (experimental conditions: Cd^2+^ concentration of 100 ppm, and transmembrane pressure of 1 bar).

Figure 5 and Figure 6 show the effect of the feed pH on the Pb^2+^ and Cd^2+^ rejection of the three membranes (i.e., PES1, PES2, and PES3) for solutions containing 100 ppm of lead and cadmium ions at a transmembrane pressure of 1 bar and temperature of 25 °C. In Figure 5, the rejection of the Pb^2+^ solution using PES1 increased from 62.4 ± 1.1% at a pH of 5.5 to 81.2 ± 2.2% at a pH of 6. In contrast, the rejection of Pb^2+^ decreased significantly with a further increase in the pH value to 6.5. Figure 5 shows that the Pb^2+^ rejection using PES2 increased from 62.45 ± 1.05% to 64.6 ± 0.6% when increasing the pH value from 5.5 to 5.7. However, there was no significant change in the Pb^2+^ rejection with further increases in the pH value from 5.7 to 6.5, and the maximum Pb^2+^ rejection was 65.2 ± 0.6% at a pH of 6.5. For PES3, there was no significant change in the Pb^2+^ rejection when increasing the pH value from 5.5 to 6.2. A further increase in the pH value decreased the Pb^2+^ rejection from 67.65 ± 0.35% at a pH of 6.2 and to 38.4 ± 1.8% at a pH of 6.5, as shown in Figure 5. 

In Figure 6, it can be observed that the Cd^2+^ rejection increased from 22.5 ± 0.5% to 63 ± 3% after increasing the pH from 5.5 to 6.5. Using PES2, the Cd^2+^ rejection increased significantly from 21 ± 3% to 44.25 ± 0.25% after increasing the pH value from 5.5 to 6.2. With a further increase in the pH value (i.e., to 6.5), the Cd^2+^ rejection decreased. For PES3, the Cd^2+^ rejection increased from 11.3 ± 0.3% at a pH of 5.5 to 30.5 ± 0.5% at a pH of 6.5, as shown in Figure 6. These results demonstrated that heavy metal rejection increased with rising pH values mainly due to the intensification of the negative charge on the membrane surface, which increased the attraction between the lead and cadmium ions and the membrane surface. Consequently, this enhanced the membrane separation performance [1,30]. Moreover, as pH increased from 5.5 to 6.5, the fiber surface charge became more negative because of the increasing OH-; therefore, precipitation of heavy metal ions occurs at the fiber surface because of the electrostatic attraction and forms a solid layer at the surface of the fiber. This solid layer can significantly affect the separation characteristics of the fiber by changing the rejection of ion metals. In this effort, it may be seen that at pH 5.5, the hollow fiber iso-electric point (IEP) is recommended. Where IEP refers to the pH value, which resulted in a less rejection of the ion. PES1, PES2, and PES3 showed normal behavior, acting as positively charged fibers at pH less than IEP and as negatively charged fibers at pH greater than IEP. Increase of rejection with increasing pH above the IEP is due to the fact that the surface of fiber becomes more negatively charged because of the increment of OH-. Therefore, adsorption of heavy metal ions occurs at the hollow fiber surface because of the electrostatic attraction, which in turn leads to an increase in rejection. Gherasim et al. [38] also recommended the IEP for their PES membrane for rejection of Cd^2+^. Also, rejection of Pb^2+^ was higher than Cd^2+^ for PES1, rejection of Pb^2+^ was higher than Cd^2+^ for PES2, and rejection of Pb^2+^ was higher than Cd^2+^ for PES3 at pH > 5.5. This can be explained due to normalized volume charge density (known as the ratio of the effective volume charge density of the membrane to the total concentration of charge in solution, expressed in equivalents of charge per volume unit). 

### 3.2. Effect of Heavy Metal Concentration on the PES Hollow Fiber Performance

**Figure 7:**
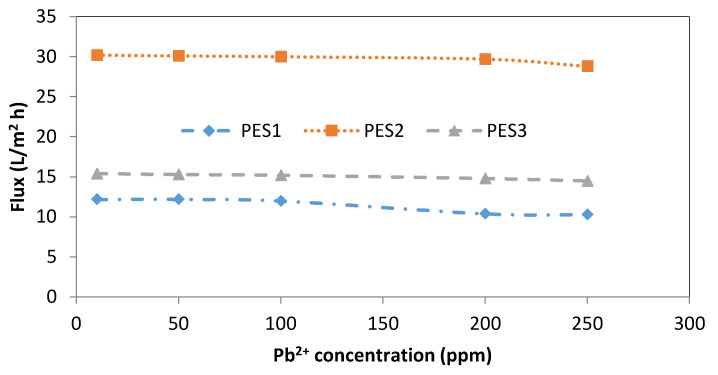
Effect of Pb^2+^ initial concentration on permeate flux of PES1, PES2, and PES3 membranes (experimental conditions: pH = 6 ± 0.2, transmembrane pressure of 1 bar).

**Figure 8:**
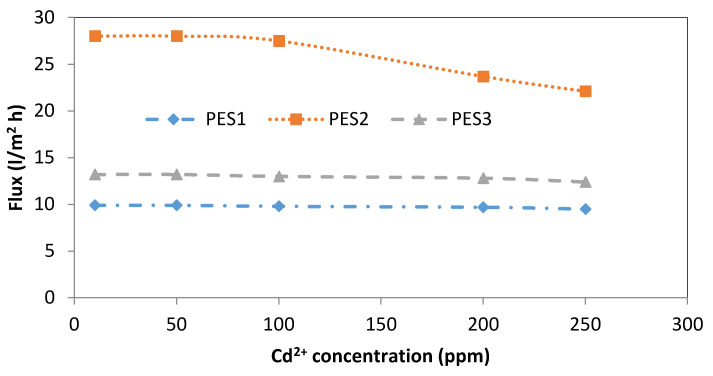
Effect of Cd^2+^ initial concentration on permeate flux of PES1, PES2, and PES3 membranes (experimental conditions: pH = 6 ± 0.2, transmembrane pressure of 1 bar).

Figure 7 and Figure 8 show the effect of heavy metal concentration on the permeation flux for three types of PES membranes at a feed temperature of 25 °C, pH = 6 ± 0.2, and transmembrane pressure of 1 bar. The permeation flux using PES1 did not change significantly when increasing the Pb^2+^ concentration from 10 to 100 ppm, whereas a 13.34% reduction in the permeation flux was observed when increasing the Pb^2+^ concentration to either 200 or 250 ppm, as illustrated in Figure 7. Moreover, Figure 8 shows that the permeation flux of only PES2 and PES3 decreased slightly after increasing the Pb^2+^ concentration to 200 or 250 ppm. In general, the permeate flux and separation factor mainly depend on the characteristics of the hollow fiber, such as pore size, distribution of the pore size at the fiber surface, and porosity, as well as the wall thickness of the fiber. Therefore, from the results of the PES membranes, it can be seen that PES2 has higher permeation flux than PES1 and PES3 due to the higher pore size and porosity and lower hollow fiber thickness, as well as wider pore size distribution, as shown in Table 1. Accordingly, it can be concluded that the performance of the hollow fiber strongly depends on the membrane properties. 

Regarding the effect of Cd^2+^ concentration on the performance of all membrane types, Figure 8 shows a minor effect of the Cd^2+^ concentration on the permeation flux of PES1 and PES3. Using PES2, the permeation flux decreased from 28 to 27.5 (L/m^2^·h) with increasing Cd^2+^ concentration from 10 to 100. While sharp decreases in the permeation flux were observed from 27.5 to 23.7 when increasing the Cd^2+^ concentration from 100 to 200, these values decreased from 23.7 to 22.1 when the Cd^2+^ concentration increased from 200 and 250 ppm, as shown in Figure 8. This decline in the permeation flux of the three types of PES fibers can be attributed to an increase in the deposition of metals on the membrane surface with increases in the heavy metal concentration. The deposition or adsorption of heavy metals on the surface of the hollow fiber resulted in a reduction of the effective pore size of the fiber.

**Figure 9:**
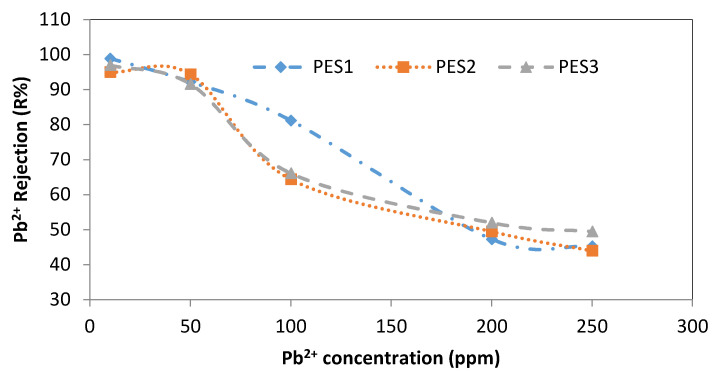
Effect of initial Pb^2+^concentration on Pb^2+^ rejection using PES1, PES2, and PES3 membranes (experimental conditions: pH = 6 ± 0.2, transmembrane pressure of 1 bar).

**Figure 10:**
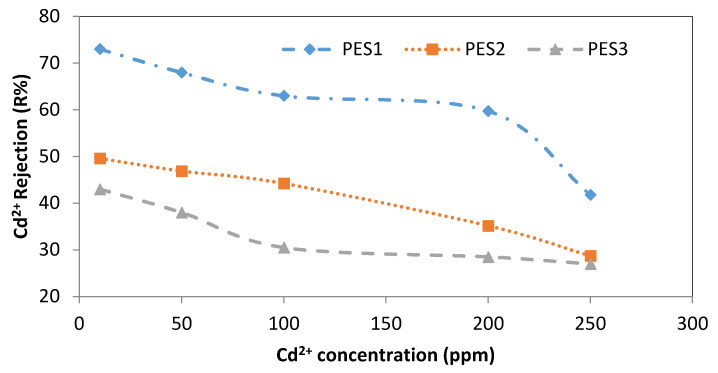
Effect of initial Cd^2+^ concentration on Cd^2+^ rejection using PES1, PES2, and PES3 membranes (experimental conditions: pH = 6 ± 0.2, transmembrane pressure of 1 bar).

The effect of various lead and cadmium concentrations (i.e., 10, 50, 100, 200, and 250 ppm) on the rejection of three PES membranes is shown in Figure 9 and Figure 10. Using the PES1 membrane, the rejection of Pb^2+^ was 98.9 ± 0.1% at 10 ppm and sharply decreased to 45.3 ± 2.25% at a concentration of 250 ppm, as shown in Figure 9. Using PES2, the rejection of Pb^2+^ was approximately constant at 95 ± 2% for the 10 and 50 ppm Pb^2+^ concentrations, whereas further increases in the Pb^2+^ concentration resulted in a sharp decrease in the Pb^2+^ rejection (i.e., 44 ± 1%), with similar behavior observed for PES3. For Cd^2+^, using PES1, the rejection at 10 ppm was 73 ± 3%, and it gradually decreased to 59.75 ± 0.4% when the Cd^2+^ concentration was reduced to 200 ppm, while using a Cd^2+^ concentration of 250 ppm, the rejection of the Cd^2+^ sharply decreased to 41.8 ± 0.2%, as shown in Figure 10. Rejection of Cd^2+^ using PES2 was 49.6 ± 0.6% at 10 ppm, while at 250 ppm, the Cd^2+^ rejection greatly decreased to 28.75 ± 1.25%. The rejection of Cd^2+^ using PES3 was 43 ± 0.9% at 10 ppm, while at 250 ppm, the Cd^2+^ rejection greatly decreased to 27 ± 0.7%, as displayed in Figure 10. 

Also, the Cd^2+^ exhibited more fouling on the membrane surface compared with the Pb^2+^. The ionic radius of the Pb^2+^ cation (i.e., 133 pm) was larger than that of the Cd^2+^ cation (i.e., 97 pm), meaning that the Pb^2+^ cation possessed a smaller hydration radius than the Cd^2+^ cation [49]. Thus, the Cd^2+^ cations had more of a tendency than the Pb^2+^ cations to attach to water molecules, stay in the solution, and pass through the membrane pores with water molecules in the permeate.

### 3.3. Estimating Parameters for the Membrane and Coefficient of Mass Transfer 

**Figure 11:**
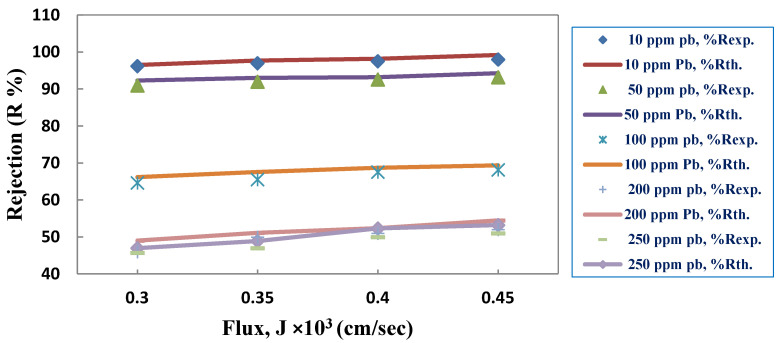
Results of CFSD model for the dataset of the PES3 membrane for Pb(NO_3_)_2._

**Figure 12:**
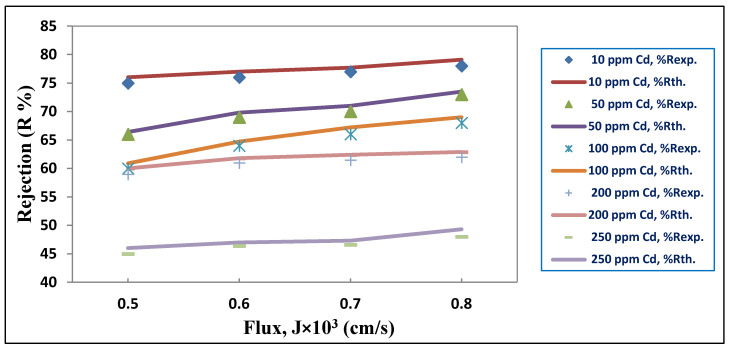
Results of CFFP model for the dataset of the PES1 membrane for Cd (NO_3_)_2._

According to the Levenberg–Marquardt method [50], the experimental data were analyzed using the SPSS version 22 nonlinear parameter estimation program, where the observed rejection (R_o_) and permeate flux (J) were calculated at conditions in which various parameters were altered (i.e., feed pH, PES membrane type, and initial ion concentration) for each dataset. The parameters estimated by applying the different models expressed by Equations (12), (20), and (22) were employed to calculate the PES membrane transport parameters and mass transfer coefficients according to their respective relations. To obtain the R_o_ of the PES membrane for different J values, these parameters were subsequently used according to the specification of each individual model, as presented in Table 3 and Table 4. PES3 was arbitrarily chosen to compare the experimental data and theoretical data of the CFSD model for Pb^2+^, while PES1 was arbitrarily chosen to compare the experimental data and theoretical data of the CFSD model Cd^2+^, as shown in Figure 11 and Figure 12. This can also be discerned through the values of the nonlinear parameters (S^2^) presented in Table 3 and Table 4, where the experimental results were substituted into the theoretical equations, and the values of the parameters were calculated. Then, these parameters were substituted again into the equations to find the second values of efficiency and J by applying a statistical program using a trial and error method. Both outputs confirmed that all of the results were equally fitted. Moreover, the model-predicted ion concentrations for specific rejection values were in good agreement with the experimental results.

The combined film theory-Spiegler-Kedem (CFSK) model showed a high degree of accuracy when applied to the experimental rejection data for all initial metal concentrations and PES membrane types. In sum, very high reflection coefficients (σ) and very low values of the permeability solute (P_s_) were obtained by fitting the CFSK model to the experimental data. As these parameters were based on the initial metal concentrations, the P_s_ increased as the initial metal concentration increased due to the high solute amount crossing through the membrane. On the other hand, a gradual decrease in the solute rejection reduction was observed with different σ, as was also found by Al-Zoubi [51]. It can be concluded that for a wide range of single-salts concentrations (e.g., 10 to 250 ppm), the model is still valid.

These results explicate the transport mechanism of solutes in these processes by the same remarks. At low pressure, a high solute transport by diffusion was expected for low rejection. At high pressure, the convective solute transport is more critical; however, this effect was not observed in the current work because the rejection was high even at low pressures. Thus, the convective transport seemed to be dominant in the rejection processes under study. Moreover, σ, a measure of the extent of the convective solute transport in the PES membranes, was almost serially hindered [52]. Therefore, the Spiegler-Kedem parameter values proved that the previous results reflected the membrane structure. Ballet et al. [26] examined the impact of Pb^2+^ and Cd^2+^ ion characteristics on the solute rejection and reported that the reflection coefficient  (σ) for each solute increased with an increase in the Pb^2+^ and Cd^2+^ ion valence, while the P_s_ decreased. Similar results were obtained by Wang et al. [53]. For the CFFP model, the effective membrane thickness (τδ/ε) can be determined from the average value of the parameter b2, which was previously calculated as 255 µm [54]. If the values of the membrane void fraction (ε) and tortuosity (τ) are assumed to be 0.16 and 3 respectively [55], the thickness of the boundary layer  (δ) will be 14, which is a reasonable value with regard to the data submitted by the supplier.

### 3.4. Estimation of the Concentration Polarization Model (CPM), Enrichment Factors (E_o_ and E), and Péclet Number (P_e_)

To calculate the true rejection using the membrane transport model, which depends on the concentration polarization, Equation (25) was applied, as it includes the factors that impact concentration polarization, namely the permeate volume flux, diffusion coefficient of the solute in the thickness of the boundary layer  (δ), and membrane enrichment factor (which depends on the C_p_/C_b_ ratio). In Table 5 and Table 6, the enrichment factors E_o_ and E for the three types of PES membranes and the solutes’ (i.e., Pb^2+^and Cd^2+^) ions are given. The concentration of the solute at the membrane surface ranged from 1.0072 to 1.0163, from 1.0177 to 1.0423, and from 1.0102 to 1.0210 times greater for Pb^2+^ and from 1.0055 to 1.0104, from 1.0076 to 1.0178, and from 1.0048 to 1.0081 times greater for Cd^2+^ than in the absence of any concentration polarization, for PES1, PES2, and PES3, respectively. With respect to reverse osmosis, the concentration polarization models are usually about 1.1 and 1.5 [52], while the E_o_ ranges from 0.0100 to 0.5100, from 0.025 to 0.57, and from 0.02 to 0.51 for Pb^2+^, and from 0.2200 to 0.5800, from 0.5 to 0.74, and from 0.56 to 0.722 for Cd^2+^ for PES1, PES2, and PES3, respectively. Regarding reverse osmosis, the enrichment factors are usually about 0.01 [52] due to the membrane solute rejection capability being about 100% [43].

The comparison between the concentration polarization and Péclet number for the PES1, PES2, and PES3 membranes at different Pb^2+^ and Cd^2+^ ion concentrations is shown in Table 5 and Table 6. When the Péclet number is large (J >> k), the convective flux through the membrane cannot be easily stabilized by diffusion in the boundary layer, and concentration polarization models will be large. On the other hand, when the Péclet number is small (J << k), the convective flux through the membrane can be easily stabilized by diffusion in the boundary layer, and concentration polarization models are close to unity [41]. The Péclet number values of the Pb^2+^ ion ranged from 0.0149 to 0.0174 for PES1, from 0.0408 to 0.0425 for PES2, and from 0.0218 to 0.0212 for PES3, and the Péclet number values for the Cd^2+^ ion ranged from 0.0131 to 0.0132 for PES1, from 0.0327 to 0.0366 for PES2, and from 0.0177 to 0.0183 for PES3. Therefore, the Péclet number is a key factor in determining the mechanism of separation by diffusion [55]. Borisov et al. [56] suggested a novel model to evaluate the impact of concentration polarization on pervaporation. They found that the intrinsic enrichment factor can be directly estimated from the experimental data. The method is also practical compared to the approaches evaluating the intrinsic membrane properties by altering the thickness of the membrane or the driving force. The influence of process parameters such as concentration of organic component, temperature, and velocity of feed solution on the membrane separation performance was studied. They found that the intrinsic enrichment factor value noticeably alters when the process parameters are changed. This change is one of the key factors affecting the value of concentration polarization modulus throughout the pervaporation process. The suggested approach provides a procedure to minimize the effect of concentration polarization on the pervaporation separation process under suitable conditions.

### 3.5. Comparative Study of PES Membranes

Table 7 depicts a comparison study between the performance of the three types of PES membranes used in the current study with the separation performance of the various types of NF membranes found in the literature. The most significant operating variables such as pH of the feed solution, feed solution concentration, and trans-membrane pressure are also presented in Table 7. It can be noticed that the three types of PES membranes’ performance in the current experimental work have excellent Pb^2+^ rejection values in comparison with most NF membranes presented in the literature. Also, it can be observed from Table 7 that the separation performance of the three types of PES membranes used in this study have a reasonable rejection of Cd^2+^ in comparison with the separation performance of most NF membranes found in the literature.

## 4. Conclusions

In the present study, three types of PES membranes, symbolized by PES1, PES2, and PES3, were used to remove the highly polluting and toxic Pb^2+^ and Cd^2+^ ions from wastewater. The performance of the membranes with single Pb^2+^ and Cd^2+^ ions, similar to those found in a real mining effluent, was evaluated. Different operating conditions were used for the removal of heavy metal ions to treat wastewater prior to discharge into the environment. It can be concluded that the permeation flux and rejection of Pb^2+^ were higher than that of Cd^2+^ at various pH values and heavy metal ions’ concentration, and that PES2 was a very efficient hollow fiber for the removal of heavy metal ions. The separation performance of the hollow fiber PES membranes strongly depends on the membrane properties, such as mean pore size, pore size distribution, and thickness. Analysis of the experimental data using CFSD, CFSK, and CFFP models showed good agreement between the theoretical and experimental results. Moreover, the active skin layer thickness and the effective membrane thickness were predicted by the CFFP model. According to the value of the Péclet number, the mechanism of separation was due to diffusion. The PES membranes’ performance in the current experimental work has excellent Pb^2+^ rejection values in comparison with most NF membranes presented in the literature.

## Figures and Tables

**Table 1:** Characteristics of the polyethersulfone (PES) membranes.

Membrane Code	Membrane Material	Average Pore Size (nm)	Pore size Distribution (nm)	Porosity (%)	Outer Diameter (µm)	Inner Diameter (µm)	Thickness (µm)
PES1	PES (29%)	52.04	25–100	52.5	1012	620	196
PES2	PES (27%)	58.11	35–130	67.6	958.4	576	191.2
PES3	PES (27%)	47.75	20–115	58.1	1005	603.6	200.7

**Table 2:** Operating conditions of the hollow fiber membrane separation process.

Metal Salts	pH _metal_	C_metal_ (ppm)	Pressure
Pb(NO_3_)_2_	5.5, 5.7, 6, 6.2, 6.5	10, 50, 100, 200, 250	1 bar
Cd(NO_3_)_2_·4H_2_O	5.5, 5.7, 6, 6.2, 6.5	10, 50, 100, 200, 250	1 bar
Pb(NO_3_)_2_ + Cd(NO_3_)_2_·4H_2_O	6 ± 0.2	10 + 50, 50 + 10, 50 + 50	1 bar

**Table 3:** Parameter estimated for various modules by a nonlinear estimated program (Pb^2+^).

Type of Membrane	No. of Set	pH	Feed Conc. (ppm)	CFSD Model		CFSK Model				CFFP Model	
				D_am_ K ^b^/ δ × 10^4^(cm/s)	k ^a^ × 10^3^ (cm/s)	σ	P_M_ × 10^5^ (cm/s)	k ^a^ × 10^3^ (cm/s)	S^2^	ε/k ^a^	εD_ab_/$\tau \delta \;\times \;10^{4}$
PES1	1	6.0	10	2.45	4.5	0.9101	5.53	20.72	0.007	7.72	9.45
	2	6.0	50	2.48	4.33	0.9080	5.97	20.53	0.009	7.79	9.27
	3	6.0	100	2.51	4.25	0.9004	6.21	20.21	0.016	7.92	9.55
	4	6.0	200	2.42	4.11	0.8864	7.84	19.73	0.032	8.11	9.8
	5	6.0	250	2.38	4.03	0.8795	8.44	19.43	0.043	8.23	10.21
PES2	6	6.5	10	2.64	4.68	0.9211	6.33	21.12	0.024	7.58	9.75
	7	6.5	50	2.58	4.55	0.9156	6.67	21.01	0.018	7.62	9.62
	8	6.5	100	2.47	4.46	0.9111	6.91	20.61	0.019	7.76	9.85
	9	6.5	200	2.38	4.43	0.8981	7.44	20.41	0.035	7.84	9.83
	10	6.5	250	2.39	4.23	0.8895	7.84	19.73	0.042	8.11	10.11
PES3	11	6.2	10	2.44	4.88	0.9255	6.43	20.92	0.00025	7.65	9.65
	12	6.2	50	2.68	4.73	0.9246	6.58	20.71	0.00049	7.73	9.41
	13	6.2	100	2.57	4.58	0.9201	6.84	20.11	0.0021	7.96	9.55
	14	6.2	200	2.78	4.54	0.9181	7.14	19.71	0.007	8.12	9.91
	15	6.2	250	2.88	4.43	0.9095	7.74	19.13	0.0059	8.36	10.31

^a^ value of mass transfer coefficient of CFSD, CFSK, and CFFP models. ^b^ Solute partition coefficient.

**Table 4:** Parameter estimated for various modules by a nonlinear estimated program (Cd^2+^).

Type of Membrane	No. of Set	pH	Feed Conc. (ppm)	CFSD Model		CFSK Model				CFFP Model	
				D_am_ K ^b^/ δ × 10^4^(cm/s)	k ^a^ × 10^3^ (cm/s)	σ	P_M_ × 10^5^ (cm/s)	k ^a^ × 10^3^ (cm/s)	S^2^	ε/k ^a^	εD_ab_/$\tau \delta \;\times \;10^{4}$
PES1	1	6.5	10	2.87	4.77	0.9227	5.58	20.98	0.048	7.63	10.23
	2	6.5	50	2.76	4.65	0.9219	6.11	20.61	0.029	7.76	10.56
	3	6.5	100	2.81	4.63	0.9205	6.54	20.33	0.057	7.87	10.66
	4	6.5	200	2.66	4.58	0.9198	7.23	20.01	0.053	8.00	10.98
	5	6.5	250	2.57	4.55	0.9187	8.67	19.88	0.074	8.05	11.32
PES2	6	6.2	10	2.97	4.87	0.9223	6.78	21.88	0.042	7.31	10.28
	7	6.2	50	2.88	4.81	0.9119	7.11	20.91	0.04	7.65	10.38
	8	6.2	100	2.84	4.67	0.9122	7.45	20.73	0.048	7.72	10.76
	9	6.2	200	2.76	4.61	0.9089	8.13	20.91	0.134	7.65	10.86
	10	6.2	250	2.51	4.53	0.9087	8.77	19.73	0.185	8.11	11.12
PES3	11	6.5	10	2.67	4.89	0.9263	6.68	20.08	0.051	7.59	10.27
	12	6.5	50	2.79	4.78	0.9219	6.01	20.95	0.112	7.64	10.67
	13	6.5	100	2.82	4.69	0.9202	7.15	20.43	0.184	7.83	10.69
	14	6.5	200	2.86	4.71	0.9189	8.03	20.11	0.254	7.96	11.58
	15	6.5	250	2.91	4.83	0.9127	8.25	19.83	0.082	8.07	11.82

^a^ value of mass transfer coefficient of CFSD, CFSK model and CFFP model; ^b^ Solute partition coefficient.

**Table 5:** Summary of CPM, enrichment factors (Eo and E), and Péclet number (Pb^2+^).

Type of Membrane	No. of Set	pH	Feed Conc. (ppm)	Enrichment Factors		CPM	k ^a^ × 10^3^ (cm/s)	Permeate Flux (×10^3^) (cm/s)	Péclet Number (J/k ^a^)
				E	Eo	C_m_/C_b_			
PES1	1	6.0	10	0.0098	0.0100	1.0163	20.72	0.361	0.0174
	2	6.0	50	0.0433	0.0440	1.0154	20.53	0.358	0.0175
	3	6.0	100	0.1648	0.1670	1.0135	20.21	0.353	0.0175
	4	6.0	200	0.4864	0.4900	1.0075	19.73	0.292	0.0148
	5	6.0	250	0.5063	0.5100	1.0072	19.43	0.289	0.0149
PES2	6	6.5	10	0.0240	0.025	1.0423	21.12	0.8972	0.0425
	7	6.5	50	0.0538	0.056	1.0402	21.01	0.8778	0.0418
	8	6.5	100	0.3585	0.368	1.0264	20.61	0.8528	0.0414
	9	6.5	200	0.5301	0.540	1.0186	20.41	0.8194	0.0401
	10	6.5	250	0.5601	0.57	1.0177	19.73	0.8056	0.0408
PES3	11	6.2	10	0.0196	0.02	1.0210	20.92	0.4444	0.0212
	12	6.2	50	0.0824	0.084	1.0196	20.71	0.4389	0.0212
	13	6.2	100	0.3493	0.354	1.0135	20.11	0.4333	0.0215
	14	6.2	200	0.4749	0.48	1.0108	19.71	0.4222	0.0214
	15	6.2	250	0.5049	0.51	1.0102	19.13	0.4167	0.0218

^a^ value of mass transfer coefficient of the CFSK and CFFP models.

**Table 6:** Summary of CPM, enrichment factors (Eo and E), and Péclet number (Cd^2+^).

Type of Membrane	No. of Set	pH	Feed Conc. (ppm)	Enrichment Factors		CPM	k ^a^ × 10^3^ (cm/s)	Permeate Flux (×10^3^) (cm/s)	Péclet Number (J/k ^a^)
				E	Eo	C_m_/C_b_			
PES1	1	6.5	10	0.2177	0.2200	1.0104	20.98	0.278	0.0132
	2	6.5	50	0.2674	0.2700	1.0097	20.61	0.275	0.0133
	3	6.5	100	0.3968	0.4000	1.0080	20.33	0.272	0.0134
	4	6.5	200	0.3868	0.3900	1.0082	20.01	0.264	0.0132
	5	6.5	250	0.5769	0.5800	1.0055	19.88	0.261	0.0131
PES2	6	6.2	10	0.4913	0.500	1.0178	21.88	0.8000	0.0366
	7	6.2	50	0.5171	0.526	1.0173	20.91	0.7806	0.0373
	8	6.2	100	0.5461	0.555	1.0163	20.73	0.7667	0.0370
	9	6.2	200	0.6735	0.680	1.0097	20.91	0.6833	0.0327
	10	6.2	250	0.7344	0.740	1.0076	19.73	0.6444	0.0327
PES3	11	6.5	10	0.5555	0.560	1.0081	21.08	0.3861	0.0183
	12	6.5	50	0.6057	0.610	1.0070	20.95	0.3806	0.0182
	13	6.5	100	0.6761	0.680	1.0058	20.43	0.3722	0.0182
	14	6.5	200	0.6963	0.700	1.0054	20.11	0.3639	0.0181
	15	6.5	250	0.7185	0.722	1.0048	19.83	0.3500	0.0177

^a^ value of mass transfer coefficient of the CFSK and CFFP models; Conc.: Concentration

**Table 7:** Comparison between the performances of the current study and types of NF membranes presented in the literature.

Type of Membrane	Module	Material Removed (Aqueous Solution)	pH	Con. ppm	Pressure	Rejection %	Ref.
NF270	Flat sheet	Pb(NO_3_)_2_/Cd(NO_3_)_2_	1.5–5	100–2000	3–5 bar	Cd^2+^ = 99% Pb^2+^ = 74%	[27]
Dual-layer NF	hollow fiber	Na_2_Cr_2_O_7_ CdCl_2_ pb(NO_3_)_2_	4.74 5.45 5.03	1000	1 bar	Cr_2_O_7_^−^ = 98% Cd^2+^ = 95% Pb^2+^ = 93%	[28]
TFC-NF300	polyamide thin film	CdCl_2_; NiSO_4_	5	5–150	2–20 atm	Cd^2+^ = 80% Ni^2+^ = 97%	[43]
ESNA1-4040	polyamide thin film	pbCl_2_	1–12	20	4–16 Mpa	pb^2+^ = 93.3%	[57]
NF (JCM)	Polyamide flat sheet, spiral wound	Pb(NO_3_)_2_ NiSO_4_	3–4	1	5.8 bar for pb^2+^ 6 for Ni^2+^	Pb^2+^ = 86% Ni^2+^ = 93%	[58]
(PEI) cross linked P84	hollow fiber	pb(NO_3_)_2_	12	1000	13 bar	pb^2+^ = 91.05%	[59]
PVDF/APTES functionalized halloysite	Flat sheet	Cu^2+^, Cd^2+^ and Cr^6+^ homogeneous solution	5.5	5	5 bar	Cd^2+^ = 44.2%	[60]
Poly/PIP PA layer modified PEI substrate: PES/Ag	Flat sheet	Pb^2+^, and Cd^2+^ solution	5.0–7.0	100	5 bar	Cd^2+^ = 97% Pb^2+^ = 99%	[61]
cellulose acetate (CA) NF-23	Flat sheet	Cd(NO_3_)_2_	2–12	0.001 mol/l	9 bar	Cd^2+^ = 84%	[62]
PES1; PES2; PES3	hollow fiber	(Cd(NO_3_)_2_·4H_2_O); (Pb(NO_3_)_2_)	5.5–6.5	10–250	1 bar	Pb^2+^ = 99%; 97.5%; 98% Cd^2+^ = 78%; 49.2%; 44%	This study

## Nomenclature

A	Membrane surface area, m^2^
b_f_	Factor measure of friction between the solute molecules and the membrane pore wall, calculated from b_f_ = 1 + f_sm_/ f_sw_
C	Solute concentration in the boundary layer, g/m^3^
C_b_	Average bulk concentration, g/m^3^
C_f_	Concentration of solute in the feed, g/m^3^
CFFP	Combined film theory-finely-porous model
CFSD	Combined film theory-solution-diffusion model
CFSK	Combined film theory-Spiegler-Kedem model
C_m_	Solute concentration at the membrane surface/water (solvent) interface, g/m^3^
CP	Concentration Polarization
C_p_	Concentration of solute in permeate, g/m^3^
CPM	Concentration Polarization Model
C_r_	Concentration of solute in retentate, g/m^3^
D_am_ K/δ	Solute transport parameter, cm/s
D	Diffusion coefficient, cm^2^/s
D_ab_	Diffusivity of solute a in solvent b, cm^2^/s
D_am_	Diffusivity of salt a on surface membrane, cm^2^/s
Eo	Enrichment Factor, known as C_p_/C_m_
F	Flow parameter defined in Equation (15)
f_sm_	Friction coefficient between solute and membrane
f_sw_	Friction coefficient between solute and solvent (water)
J_S_	Solute flux through membrane, m^3^/m^2^s
J_v_	Convective + Diffusive mass transfer rate, m^3^/m^2^s
K	Solute partition coefficient
k	Mass transfer coefficient expressed as $k=\frac{D_{ab}}{\delta }$
P_e_	Péclet number (a dimensionless number)
P_M_	Salt permeability, L/m^2^·h
P_s_	Overall permeability coefficient
PWP	Pure water permeability
R	True solute rejections
R_exp_	Experimental rejection
Ro	Observed rejection
R_th_	Theoretical rejection
t	Collected permeate time, h
TMP	Transmembrane pressure, bar
V	Permeate volume, L
x	Distance from the membrane layer, m

## Greek Letters

ε	Membrane void fraction
τ	Tortuosity
δ	Layer thickness; thickness of the boundary layer, m
$\left(\frac{\tau \delta }{\varepsilon }\right)\;$	Effective membrane thickness
ΔP	Transmembrane pressure, bar
Δπ	Osmotic pressure difference, bar
